# Camera-based mobile applications for movement screening in healthy adults: a systematic review

**DOI:** 10.3389/fspor.2025.1531050

**Published:** 2025-05-09

**Authors:** Inaam El-Rajab, Thomas J. Klotzbier, Heide Korbus, Nadja Schott

**Affiliations:** Department of Sport and Exercise Science, University of Stuttgart, Stuttgart, Germany

**Keywords:** exercise, motion analysis, mhealth, ehealth, health-related, mobile application

## Abstract

**Background:**

In recent years, the proliferation of mobile applications in the health and fitness sector has been rapid. Despite the enhanced accessibility of these systems, concerns regarding their validation persist, and their accuracy remains to be thoroughly evaluated compared to conventional motion analysis methodologies. Furthermore, there is a paucity of evidence regarding real-time feedback and movement quality assessment. Consequently, this systematic review aims to evaluate the current state of camera-based mobile applications for movement screening in healthy adults, focusing on specific types of movement.

**Methods:**

A systematic literature search was conducted in four databases—PubMed, ScienceDirect, Web of Science, and IEEE Xplore—covering the period from 2000 to 2024. The search strategy was based on key terms related to four main concepts: screening, mobile applications, cameras, and physical activity. The Preferred Reporting Items for Systematic Reviews and Meta-Analyses (PRISMA) guidelines were followed. The study was registered *a priori* on PROSPERO (Registration ID: CRD42023444355) to ensure transparency and prevent selective reporting of outcomes.

**Results:**

Of the 2,716 records initially identified, eight studies met the specified inclusion criteria. The studies were primarily concerned with fitness exercises, gait analysis, and sport-specific movements. Some studies demonstrated high reliability compared to gold standard systems, while others reported technical limitations such as camera positioning and data interpretation issues. Feedback mechanisms varied, with many applications lacking personalized real-time correction.

**Conclusion:**

Despite the potential of smartphone-based movement screening applications, particularly their accessibility and affordability, challenges remain regarding accuracy and user feedback. Precise measurements comparable to established methods are crucial for application-oriented camera-based movement screening. Equally important are improving real-time feedback, expanding the types of movement that can be assessed, and ensuring broad applicability across different populations and environments to ensure sustainable use of application-based movement screening.

## 1 Introduction

The presence of mobile applications, including those pertaining to the sports/fitness/health market, has accelerated markedly in recent years (1). The most popular mobile application marketplaces offer users millions of applications (Google Play Store: 2.3 million apps; Apple App Store: approximately 2 million) (2). By 2024, more than 200,000 health and fitness applications had become available across various app stores worldwide (3). These applications address a comprehensive range of user needs, encompassing general fitness and wellness and more specific areas such as medical management and health behavior changes (4). Most users, 517 million, opt for free fitness applications, while a smaller number, 384 million, choose paid applications (5). Fitness and Exercise applications that provide support and guidance for fitness workouts and incorporate gamification elements are highly popular due to their ability to be used on demand, aligning with the current market for flexibility (6, 7).

Despite the growing prevalence of medical and exercise applications, there is a paucity of studies that assess the accuracy and efficacy of camera-based mobile applications used for movement assessment and exercise guidance, and their findings are subsequently published in peer-reviewed journals. A few of these studies have been summarized in previous systematic reviews. For example, Thompson et al. (8) investigated mobile applications to support therapeutic exercises targeting muscle pain and demonstrated that such applications may effectively reduce pain levels. In a similar therapeutic context, Pfeifer et al. (9) analyzed the effectiveness of mobile interventions in patients with chronic pain and concluded that these interventions can be beneficial in reducing pain. Nussbaum et al. (10) conducted a systematic review of mobile health applications in rehabilitation and found that these applications demonstrated good psychometric properties when measuring specific physical activity or gait parameters. Furthermore, when used as interventions, they positively affected various medical and functional outcomes. While these reviews provide important insights into therapeutic use cases, few have systematically examined the diagnostic validity of movement-focused applications under gold-standard conditions. Other reviews, such as those by Moreira et al. (11) and Milani et al. (12), examined mobile applications for postural assessment. However, they primarily addressed static analysis and did not consider the dynamic aspects of human movement, which are central to the scope of our review. Among the existing reviews on the use of mobile applications in the context of human movement, the work by Silva et al. (13) is notable for its focus on the validity and reliability of applications designed to assess force, power, speed, and change of direction. However, their review considers mobile applications more broadly without distinguishing smartphone-based applications specifically. Moreover, their study does not address the potential role of feedback provided by these applications, an aspect that represents a crucial gap in the current literature.

In the absence of robust validation, users may receive ineffective or even detrimental recommendations, particularly when engaging in unsupervised physical activity (see the discussion of harmful applications by (14). Those applications typically lack a crucial feature: the ability to correct user movements during exercise (15). Incorrect movements can cause pain and injury, discouraging physical activity and leading to further deterioration of health conditions. Fitness applications that analyze movement and tailor recommendations can help prevent this vicious cycle. When adequately validated, such applications could offer substantial benefits, especially in contexts lacking professional supervision.

Most highly validated movement analysis systems are characterized by high time demands, significant costs, and limited accessibility, typically confined to clinical or research settings (16). Consequently, these systems are not accessible to the typical consumer. The current gold standard for human motion analysis are optical 3D motion capture systems, which employ multiple cameras and markers on the moving body. Emerging technologies, such as markerless systems utilizing devices such as the Kinect, have been developed as more affordable alternatives, particularly in the domains of physical therapy and rehabilitation [(17); see also (18)]. Similarly, advances in smartphone technology have created opportunities for movement screening and analysis via mobile devices, offering greater accessibility, convenience, and the potential for direct, real-time feedback. However, there is a paucity of mobile applications with camera-based movement screening, where a person's movements are captured via a mobile device and analyzed in real-time. Furthermore, these applications are poorly represented, validated, and rarely peer-reviewed or presented in scientific journals. Thus, our aim is (1) to identify current camera-based mobile applications, (2) to examine which movement skills are addressed and whether these tools provide immediate feedback to the user regarding movement quality, and (3) to highlight areas for further improvement and validation, with a particular focus on enhancing measurement accuracy and usability—factors for the effective everyday use of camera-based movement analysis systems.

We provide an overview of the various applications and the movement skills under investigation. While reviews already exist that focus on rehabilitation, the scope of this work is on applications for healthy individuals without motor and/or cognitive limitations. From a movement science perspective, the technical implementation and the underlying algorithms for motion analysis are not the primary focus. This review focuses on camera-based movement analysis applications that deliver immediate results without post-processing and are compatible with standard mobile devices such as smartphones or tablets. By presenting an evidence-based overview of apps validated in scientific studies, the review offers practical value. It helps distinguish between tools supported by empirical data and those still in early development, guiding practitioners and researchers toward reliable and accurate solutions for practical use.

## 2 Methods

### 2.1 Protocol and registration

This systematic review was conducted in accordance with the Preferred Reporting Items for Systematic Reviews and Meta-Analyses [PRISMA, Page et al. (19)]. It was registered *a priori* with PROSPERO (Registration ID: CRD42023444355) to ensure transparency and prevent selective reporting of outcomes.

### 2.2 Eligibility criteria

The study focused on the availability of full-length articles. We considered original research articles published in peer-reviewed journals and conference papers in English or German between 2000 and 2024. The participants targeted in the included studies were adults aged 18 years or older. All participants were required to be healthy and to have no motor-cognitive disabilities or other disorders/disabilities to exclude studies focusing on rehabilitation contexts. A mandatory inclusion criterion was the utilization of a movement screening methodology through camera-based mobile applications, with the supplementary requirement that the live and real-time movement analysis constituted an intrinsic component of the application rather than merely recording the movement for subsequent examination through disparate software.

### 2.3 Literature search

A comprehensive search was conducted using the following databases: PubMed, Science Direct, Web of Science, and IEEE Xplore. The search was conducted on September 28, 2023, and updated on February 22, 2024. Studies published from the year 2000 onwards were included in the search. The search strategy consisted of key terms (MeSH terms in PubMed) relevant to four key concepts: screening, mobile application, camera, and physical activity. The entire electronic search strings across the various databases is presented in Table 1.

**Table 1:** Overview of database-specific search strategies and search strings.

Database	String
Pubmed	(assessment OR screening OR diagnos* OR examination) AND (mobile* OR “mobile application*” OR “mobile phone” OR “mobile device*” OR “mobile health” OR “mobile technolog*” OR smartphone OR “cell phone” OR digital* OR “digital technolog*” OR “digital health application*” OR Tablet OR ehealth OR mhealth) AND (camera* OR video*) AND (movement OR “physical activity” OR exercise OR training OR fitness OR “physical fitness”) NOT (disease OR disorder)
ScienceDirect	(assessment OR screening) AND (“mobile application” OR mhealth) AND (camera) AND (movement OR “physical activity” OR exercise) NOT (disease)
Web of Science	(assessment OR screening OR diagnos* OR examination) AND (mobile* OR “mobile application*” OR “mobile phone” OR “mobile device*” OR “mobile health” OR “mobile technolog*” OR smartphone OR “cell phone” OR digital* OR “digital technolog*” OR “digital health application*” OR Tablet OR ehealth OR mhealth) AND (camera* OR video*) AND (movement OR “physical activity” OR exercise OR training OR fitness OR “physical fitness”) NOT (disease OR disorder)
IEEE Xplore	(assessment OR screening OR diagnos* OR examination) AND (mobile* OR “mobile application*” OR “mobile phone” OR “mobile device*” OR “mobile health” OR “mobile technolog*” OR smartphone OR “cell phone” OR digital* OR “digital technolog*” OR “digital health application*” OR Tablet OR ehealth OR mhealth) AND (camera* OR video*) AND (movement OR “physical activity” OR exercise OR training OR fitness OR “physical fitness”) NOT (disease OR disorder)

Note: We specifically included the term “diagnosis” in the search strategy to broaden the scope of the search and to ensure that potentially relevant studies that did not have direct diagnostic applications but could still be relevant to the topic of the investigation were included.

### 2.4 Identification and selection of studies

The studies identified from the various databases were recorded, and their metadata was exported to a Microsoft Excel spreadsheet. The dataset comprises a variety of fundamentals, including author(s) name(s), year, journal name, title, and so forth. Duplicates were removed. Two of the three reviewers (IE-R and HK) evaluated each title and abstract for potential eligibility using pre-established criteria based on the eligibility criteria described above. If an article was initially deemed suitable for inclusion, the full text of the remaining paper was assessed. Authors of articles were contacted via email if the full-text manuscript was unavailable. All three reviewers independently screened each full-text article against the eligibility criteria (IE-R, TK, and HK). Any conflicts during screening were resolved through discussion between the three reviewers. The included studies can be found in Table 2.

**Table 2:** Overview of included studies on camera-based mobile applications for movement screening.

First author(s), year; country	Study aim	Sample (*n*; population; age)	Technical implementation (software/hardware)	Movement skill	Type of feedback/coaching	Reference standard	Main outcome
Fanton and Harari (2022) (20); USA	Validation of a functional movement assessment	*n* = 150; age = 18–85 years; ♂ = 56 ♀ = 94	Software: Halo Movement App (HMA) Hardware: Smartphone (not further specified)	Single leg stance, forward lunges, overhead squat, overhead reach, feet together squat	Score 0–100 (100 = best) based on computer vision algorithms applying deep neural networks; no feedback to users on the quality of movement performance	-Xsens sensors -13 standardized functional movement tests	Moderate to strong correlations between HMA overall score, sensor-based 3D motion capture metrics, and scores from 13 standardized functional movement tests Ability to differentiate regular healthy individuals from professional athletes and impaired participants
Tran (2020) (21); USA	Developing an application to track and calculate 3D lower-body gait in real-time	n.a./case study	Software: LGait—Apple ARKit-3 (Lower-Body Motion Tracking version 1.0.1) Hardware: iPhone 11, iPad mini 5, and iPad Pro	Gait	Measurement of joint angles, but no feedback on the quality of the gait or information on possible improvements	Vicon Motion System	Angle values are compatible (differences in the angles between the two systems are about 2^◦^), but the LGait application slightly delays tracking the gait cycle
Pham (2022) (22); Vietnam	Developing an automated system to recognize and evaluate physical exercises	*n* = 9 (5 for training, 4 for testing); age = 15–55 years	Software: Google MediaPipe Hardware: Smartphone (not further specified)	Arm circles, squats, and standing crossover toe touches	Depending on the deviation of individual joint movements, a score is calculated that gives the user feedback on what should be improved	n.a.	Average accuracy of 98.33% in recognizing movement skills
Aoyagi (2022) (23); Japan	Developing an application that enables markerless 3D motion capture	*n* = 90 original humanoid computer graphics (CG) characters created (VRM format)	Software: Three-Dimensional Pose Tracker for Gait Test (TDPT-GT) Hardware: iPhone 12 and iPhone SE2	Gait	so far, no feedback for users	Vicon Motion System	Application reconstructs the 3D full-body human motion efficiently in real time
Fernandez (2023) (24); New Zealand/Philippines	Validation of a computer-vision-based application with lab-based systems to quantify calf raise outcomes	*n* = 13 ♂ = 6; age = 38 (10) years ♀ = 7; age = 34 (7) years	Software: Calf Raise App (CRapp) Hardware: Two iPad Air 2 devices (Apple, iOS 14.1)	Calf raises	Videos for raters, but no feedback on performance for users	-3D Motion Capture System -Force Plate	Good to excellent validity across measures and excellent intra- and inter-rater reliability
Stanton (2017) (25); Australia	Examine the concurrent validity and intra-rater reliability of the MyJump app compared to laboratory-based measurements	*n* = 29; age = 26.41 (5.36) years ♂ = 10 ♀ = 19	Software: MyJump App Hardware: iPhone 6s	Counter Movement Jump (CMJ) and Drop Jump (DJ)	Provides jump height based on flight time; no feedback on quality of movement execution	Force plate	MyJump is valid and highly reliable for CMJ and DJ performance measurement and has a strong correlation for CMJ and DJ with force plate data
Jeon (2021) (26); Korea	Optimization of 2D human pose estimation for mobile devices with real-time feedback	*n* = 23; age not specified	Software: TensorFlow Hardware: Samsung S10, Samsung Note9, Google Pixel 3, iPhone 11	Chest-stretch, squat shoulder press, tuck jump, side-bend knee-up, and barbell power clean	Feedback on deviations in real-time based on an action database	COCO dataset (Common Objects in Context)	Average precision of 65.2% (COCO), 89.6% (Fitness dataset) and consistent detection of joint coordinates, 97.39% accuracy in movement counting
Li (2021) (27); Taiwan	Developing a system for the analysis of baseball swings and distinguishing good from bad swings	*n* = 10; age not specified ♂ = 10	Software: Open Pose Hardware: Smartphone (not further specified)	Baseball swing	Overall score from 0 to 100, based on custom rules	Baseball swing distance	The system's score positively correlates with hit distance, indicating its accuracy in distinguishing good and poor swings

Abbreviations: n.a., not applicable.

### 2.5 Data extraction

The following key data were extracted from each study: first author(s), year, country, study aims, sample characteristics (e.g., age, gender, health status), movement screening methods, quality criteria, outcome measures, and limitations. Three independent reviewers extracted the data to minimize errors and bias. Any discrepancies were resolved through discussion. The extracted data were then compiled into a summary table, which formed the basis for the descriptive analysis and synthesis of results.

### 2.6 Study risk of bias assessment

The studies included in this review are highly heterogeneous in content and research design, which limits the applicability of standard quality assessment tools and complicates direct comparison. Existing instruments, such as the JBI Critical Appraisal Tools, did not match the methodological characteristics of the included studies. Specifically, the checklists designed for diagnostic accuracy and analytical cross-sectional studies were inappropriate for this review. Furthermore, a search within the EQUATOR Network for alternative checklists did not yield any tools that would provide meaningful added value for assessing study quality from a movement science perspective. To maintain focus on the primary objectives of this systematic review, we chose not to conduct a formal quality assessment using conventional appraisal tools. The aim of this review is not to evaluate the methodological rigor or the effectiveness of interventions. As a result, a formal risk of bias assessment was not conducted.

## 3 Results

### 3.1 Study selection

**Figure 1:**
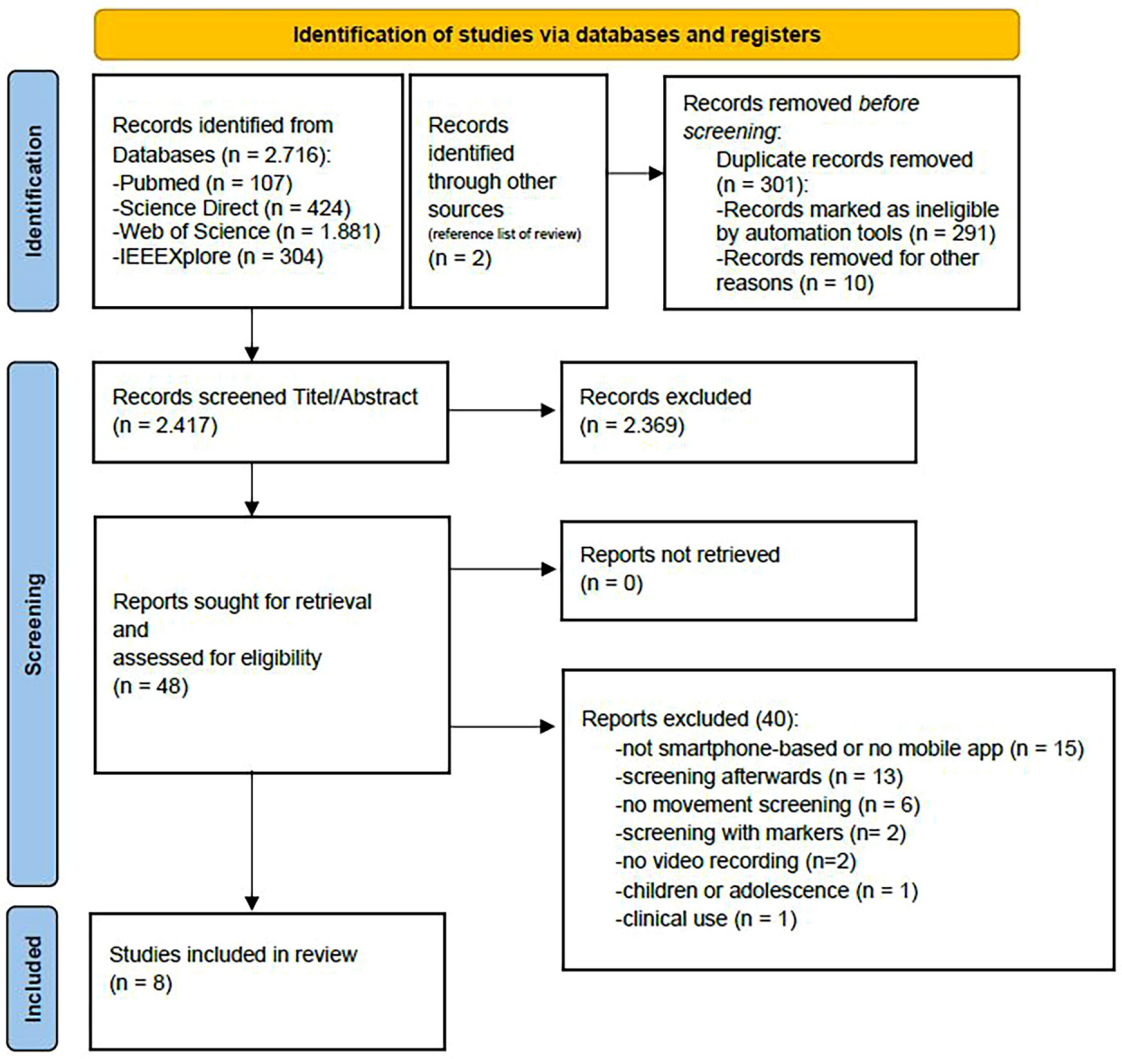
Flowchart of the study selection process, according to the PRISMA guidelines (19).

In the initial search, 2,718 entries were identified across the following databases: PubMed (107), Science Direct (424), Web of Science (1,881), and IEEE Xplore (304). Two additional entries were identified through the reference list of a review paper (15). Subsequently, 301 entries were identified as duplicates and removed. The remaining 2,417 records were assessed based on their title and abstract, excluding 2,369 studies that did not meet the pre-established inclusion criteria. Following this initial screening, 48 full-text reports were selected for further evaluation. In the subsequent detailed assessment, 40 reports were excluded for various reasons. Some studies were not based on smartphone or video technologies, while others focused on screening methods that were applied retrospectively. Additionally, studies employing marker-based tracking and those lacking video recording were excluded. One study was excluded because it focused on children or adolescents, and another was excluded because it was used exclusively in a clinical setting (see Figure 1). Following this review process, eight studies were included in the final analysis of this systematic review.

### 3.2 Characteristics of studies

The studies were conducted in various countries, including the USA (*n* = 2), Vietnam, Japan, Korea, Taiwan, New Zealand, and Australia. They were conducted between 2017 and 2023, although the inclusion criteria allowed for studies published since 2000. This suggests that research in this area has gained momentum only in recent years.

The included studies aimed to validate mobile applications for tracking and assessing specific movement patterns such as jumps (25), gait (21, 23), sport-specific movements (27), and fitness exercises (20, 22, 24, 26). The studies examined how accurately these mobile systems work compared to laboratory-based reference standards and whether they can analyze movements effectively in real-time.

The populations under investigation exhibited considerable diversity, although they often comprised relatively limited sample sizes. For example, Fanton and Harari et al. (20) collaborated with a diverse cohort of 150 individuals aged 18–85, encompassing various body types and performance levels. In contrast, Pham et al. (22) focused on a considerably smaller sample of nine participants aged between 15 and 55. Other studies, such as those by Aoyagi et al. (23), did not involve human participants but original humanoid computer-generated figures. Tran et al. (21) conducted a case study with only one participant.

Smartphone-based applications employing sophisticated technologies such as Apple ARKit-3 (21), Google MediaPipe (22), and OpenPose (27) were utilized to monitor and evaluate movement capabilities in real-time. In all cases, motion capture was conducted without the use of markers through the application of AI-driven software. Jeon et al. (26) employed TensorFlow for real-time human pose estimation. In contrast, Aoyagi et al. (23) utilized a specialized motion-tracking application, TDPT-GT, which facilitated markerless whole-body gait analysis with AI-based algorithms. The motion monitoring methods employed in the applications are based on computer vision and machine learning to track and analyze human movements. The technical implementation, including the specific software and hardware used in each study, is presented in Table 2. However, since this review has been compiled from a movement science perspective, the technical aspects are not discussed in detail.

The included studies examined fundamental movement patterns or sport-specific exercises intending to assess various aspects of physical performance. Fanton and Harari et al. (20) focused on functional movements encompassing the three primary movement criteria: mobility, stability, and posture. This was achieved by utilizing the functional movements of a one-leg stance, forward lunge, overhead squat, overhead reach, and squat with feet together. The focus on these functional movements, which are essential for daily life and overall physical well-being, aligns with the study's objective of quantifying the performance of activities of daily living in the general population. This approach provides a practical and accessible method for monitoring physical capabilities, avoiding the need for specialized equipment. Similarly, Pham et al. (22) and Jeon et al. (26) evaluated fitness exercises. The authors justified their selection of movements by stating that these exercises can be performed without professional assistance. They also noted that feedback and assessments can be provided easily. This was particularly important during the global COVID-19 pandemic of 2020, when a significant proportion of the population was required to exercise at home. Pham and colleagues (22) sought to address the issues associated with incorrect form and technique, potentially leading to muscle strain or injury. The studies by Tran et al. (21) and Aoyagi et al. (23) focused on the development of cost-effective and accessible solutions for human gait analysis, recognizing its importance in assessing physical health. The distinction between these two studies is that Aoyagi et al. (23) employed a comprehensive whole-body approach to assess general movement patterns and postural control during walking. In contrast, Tran et al. (21) focused on a detailed joint-specific analysis of the lower body (hip, knee, ankle). Similarly, Fernandez et al. (24) also focused on the lower body, with a particular emphasis on the calf, utilizing the calf raise test (CRT). The studies conducted by Stanton et al. (25) and Li et al. (27) examined movements specific to athletic performance. Stanton et al. (25) examined the counter-movement jump (CMJ) and drop jump (DJ), while Li et al. (27) examined the baseball swing. The movement skills assessed in these studies illustrate the broad range of physical abilities and performance metrics these applications can capture and analyze.

The type of feedback also exhibited considerable variability across the studies. In the Halo Movement application by Fanton and Harari et al. (20), feedback is provided as a score ranging from 0 to 100, with 100 representing the optimal score. A similar numerical rating system was employed in the studies by Li et al. (27) and Aoyagi et al. (23). In the study by Li et al. (27), feedback on the baseball swing is provided through an individualized scoring system based on biomechanical rules. The system captures essential limb coordinates during the swing, including hip distance and limb angles, utilizing the OpenPose model. Subsequently, these measures are compared against predefined custom rules derived from prior research and coaching experience. The scoring system developed by Aoyagi et al. (23) is based on calculating gait parameters, providing a quantitative assessment of the gait pattern. The present study's objective is to establish the system as a screening tool by detecting anomalies in gait behavior that could indicate the presence of movement disorders. A similar approach is employed in the study by Tran et al. (21). The recorded data are presented as 3D angular values, which allows for assessing movement patterns that experts can interpret. However, the authors do not provide any specific information on personalized feedback or coaching mechanisms.

The remaining studies included in this review (22, 24–26) similarly lack descriptions of personalized instructions or detailed correction suggestions. Therefore, the feedback is quantitative and based on the accuracy of movement detection. However, it does not provide customized instructions to improve exercise techniques. In Fernandez et al. (24), the feedback is intended for interpretation by researchers or clinicians. In contrast, the application developed by Pham et al. (22) offers supportive guidelines to users. The application developed by Jeon et al. (26) offers a quantitative assessment of movement deviation, expressed as numerical values that quantify the discrepancy between actual joint dynamics and the ideal model. A low numerical value indicates a significant deviation from the reference value. In the study by Stanton et al. (25), the MyJump application was utilized to quantify the jump height based on the flight time calculated from the moment of take-off to landing. Thus, the MyJump application provides real-time feedback on the jump height, thereby offering a readily accessible method for users to monitor their performance. In summary, the primary objective of most studies was to assess the validity and reliability of the applied methods. Most of these studies primarily evaluate performance based on quantitative scores, neglecting to provide qualitative feedback on movement execution. Consequently, the paucity of studies has resulted in a lack of personalized recommendations for performance enhancement.

The results of the reference standards exhibit considerable variation across the included studies. The employment of reference standards serves the objective of evaluating the efficacy of motion analysis in mobile systems. Fanton and Harari et al. (20) used Xsens sensors and 13 standardized functional movement tests as reference standards. Correlations between Halo Movement scores and sensor-based performance metrics ranged from 0.23 to 0.83 (*p* < 0.05), demonstrating a statistically significant relationship between the application output and objective sensor data. Additionally, the correlation between the Halo Movement scores and the thirteen clinically validated functional movement tests ranged from 0.29 to 0.63 (*p* < 0.05), indicating moderate to strong agreement. Furthermore, the system demonstrated the capacity to effectively differentiate between healthy individuals, professional athletes, and participants with impairments, as evidenced by the intrasubject coefficient of variation (1.64 ± 1.42%).

Two research groups have compared their gait analysis applications with the Vicon Motion System (21, 23). In the study by Tran et al. (21), the primary statistical outcome was the comparison of angle measurements between the LGait application and the Vicon system, with a particular focus on hip flexion and extension angles. The discrepancy in angles between the two systems was reported to be approximately two degrees, demonstrating a high degree of concordance between the mobile application and the Vicon system. However, the LGait application exhibited a minor delay in tracking the gait cycle. The TDPT-GT application by Aoyagi et al. (23) and the Vicon system were calculated for various joint measurements during rotational movements. The Pearson's correlation coefficients between the reference standard and the application vary across different joints. The findings suggested that the TDPT-GT application could adequately capture three-dimensional joint coordinates. However, specific axes demonstrated lower correlations, underscoring certain constraints in terms of precision.

Two studies utilized the force plate as a reference standard (24, 25). In the study conducted by Fernandez et al. (24), the force plate was employed to validate two-dimensional motion tracking to measure the kinetics associated with a calf raise test. In contrast, in the study by Stanton et al. (25), the force plate was utilized to validate jump height measurement by employing time-of-flight recordings. The results of the study by Stanton et al. (25) demonstrated good to excellent validity and excellent intra- and interrater reliability. The correlation between the MyJump application and the force plate for jump height measurement was *r* > 0.99 (*p* < 0.001) for both the CMJ and DJ, indicating near-perfect agreement between the two measurements. The intraclass correlation coefficients (ICC) for evaluating intrarater reliability were 0.99 (95% CI: 0.98–0.99) for the CMJ and 0.99 (95% CI: 0.60–0.99) for the DJ, indicating excellent consistency between the measurements. In addition, the Bland-Altman analyses for the CMJ demonstrated minimal dispersion around the mean, with most measurements falling within the 95% confidence limits, confirming good agreement without significant systematic bias. A slight systematic bias was observed in the DJs, with higher jump heights tending to be measured with the force plate rather than the application [*t*(26) = −10.02, *p* < 0.01]. In the study by Fernandez et al. (24), the force plate and the 3D motion capture system were used as a reference standard to record the kinetics and kinematics of the calf raise concurrently. The 3D motion capture system was utilized to accurately quantify movement kinematics, focusing on the marker positions on the lateral malleolus and heel, which mapped the spatial movement of the joints. On the other hand, the force plate was employed to record ground reaction forces, which were subsequently utilized to calculate kinetic parameters such as peak force, positive and negative work, and fatigue index. The findings revealed a high degree of concordance between the Calf Raise application (CRapp) and the reference system, particularly in the kinematics of the lateral malleolus (ICC: 0.963–1.00). However, for certain kinetic measurements, such as heel displacement and fatigue index, the coefficient of variation for the fatigue index at the lateral malleolus was 15.3% and at the heel 33.3%, indicating a lower precision. Consequently, Fernandez et al. (24) have validated the validity and reliability of the CRapp for evaluating the outcomes of the calf raise test in healthy adults.

Two studies did not use any traditional reference system. The experiment by Li et al. (27) showed a positive correlation between the swing score and stroke distance, indicating that the scoring method differentiates between swings of varying quality. Research has demonstrated a consistent correlation between higher scores and longer swing distances. Several significant biomechanical factors, including hip width and the angles of the arms and legs, determine the swing's quality. However, the authors did not provide statistical values. Jeon et al. (26) used a fitness motion database [Microsoft COCO: Common Objects in Context; (28)], which contained pre-recorded fitness actions with ideal joint positions as a reference for comparing user movements. The application utilizes a numerical score to calculate the user's joint dynamics and then compares it to the database. The application demonstrated a 97.39% accuracy rate in identifying fitness exercises, correctly recognizing 560 out of 575 movements. A closer examination of the model reveals an average precision of 65.2% on the Microsoft COCO dataset and an improved average of 89.6% on the fitness dataset. This finding underscores the model's capacity for adaptation and enhancement in recognizing particular fitness movements. Notably, Pham et al. (22) did not provide an external reference standard. The reported accuracy of 98.33% refers to internal classification performance using a trained machine learning model, rather than validation against an external reference standard. As such, this metric reflects the model's internal effectiveness but should be interpreted with caution when assessing validity.

The study results indicate that smartphone-based applications can potentially serve as a promising and practical tool for movement screening. The validity and reliability of these applications have been demonstrated to be comparable to that of established reference standards, such as motion capture systems and force plates. This comparison has mainly focused on assessing fundamental fitness exercises, gait, and sport-specific movements. However, the study populations varied in age, physical ability, and size, and many samples were relatively small, which may limit generalizability. Furthermore, although quantitative feedback is frequently offered, personalized and qualitative feedback concerning movement execution remains deficient in most applications, underscoring a pivotal domain for future development.

### 3.3 Limitations of the included studies

A range of limitations in different categories were examined in the studies. As evidenced by studies such as those conducted by Aoyagi et al. (23) and Fanton and Harari et al. (20), the systems utilized exhibit limited validation and reliability. Aoyagi et al. (23) emphasize that the application's capacity to monitor a single individual simultaneously necessitates high-quality video recordings. Fanton and Harari et al. (20) have also criticized the lack of validation for sub-values of mobility, stability, and posture, which are crucial for a comprehensive understanding of physical performance. Identifying specific weaknesses or areas for improvement becomes challenging without the validation of these components. As Stanton et al. (25) have observed, intra-rater reliability is contingent upon the expertise of the users. The incorporation of substantial measurement errors by novice users further restricts the generalizability and practical application of the findings. It is imperative to acknowledge the limitations of the study by Fernandez et al. (24), which exclusively included healthy adults, thereby constraining the external validity of its findings to other demographic groups, such as older adults, children, or individuals afflicted with chronic diseases. Additionally, Fanton and Harari et al. (20) underscore the constrained sample size and absence of heterogeneity, which further restricts the generalizability of the results.

Technical limitations include difficulties in capturing small joint segments (21), the necessity for high-quality video recordings (23), and challenges due to disparate camera positions and possible vibrations (25). Li et al. (27) underscored the significance of incorporating additional critical elements specific to movement, such as timing, swing speed, and stance during a baseball swing, into the analysis. This oversight may compromise the precision of the findings, as these elements are indispensable for performance assessment. Li et al. (27) observed that achieving a high score does not guarantee optimal performance, underscoring the necessity of incorporating movement quality metrics. Pham et al. (22) proposed expanding the range of exercises the system can recognize, aiming to enhance both applicability and user engagement. In this regard, Fanton and Harari et al. (20) mentioned the potential for over- or under-representation of specific movement patterns, which may be influenced by the system's reliance on a movement database to compare and analyze these movements (26). Some studies emphasized the necessity for enhancements in user feedback and movement quality. Pham et al. (22) suggested implementing supplementary assessments to offer more detailed feedback, allowing users to make specific improvements and identify areas that need attention.

A comprehensive review of the extant literature reveals significant shortcomings in the studies reviewed. These shortcomings pertain to the validity and generalizability of the studies, the technical requirements of the studies, and the movement analysis in the studies. The shortcomings highlight the necessity for more robust validation, enhanced adaptability to diverse user groups, and improvements in system functionality. These improvements are crucial to ensure more accurate and comprehensive performance assessments, especially regarding feedback.

### 3.4 Risk of bias assessment

The studies demonstrate a substantial degree of variation in the level of detail provided regarding the recruitment of participants. The studies conducted by Fernandez et al. (24), Fanton and Harari et al. (20), and Stanton et al. (25) provide comprehensive descriptions of their recruitment processes and inclusion criteria, thereby enhancing the internal validity of their results. Conversely, studies such as Aoyagi et al. (23), Jeon et al. (26), Tran et al. (21), and Pham et al. (22) prioritize technical aspects and provide minimal information on recruitment, which raises concerns about potential selection bias. Tran et al.'s (21) study is a case study involving a single participant, which limits the generalizability of the results compared to the other studies. In contrast, the other studies avoided a case-control design by recruiting from broader populations or activity groups without medical pre-selection, enhancing their external validity.

Most studies ensured that all participants were included in the analysis, contributing to reliable results by minimizing the potential for bias from excluding specific individuals. In contrast, Fernandez et al. (24) included all participants who completed the CRT sessions, while Fanton and Harari et al. (20) included all 150 participants, even those who could not complete all tasks. However, Tran et al. (21), Pham et al. (22), and Aoyagi et al. (23) did not provide adequate details on the inclusion criteria for participants, which may have resulted in ambiguity and potential bias in the research findings. The number of participant exclusions was minimal. The exclusion of participants with injuries by Fernandez et al. (24) was justified. Stanton et al. (25), Pham et al. (22), Aoyagi et al. (23), and Jeon et al. (26) did not specify exclusion criteria. Fanton and Harari et al. (20) endeavored to ensure diversity within the study, yet due to safety concerns, certain participants were excluded. Stanton and colleagues (25) documented two exclusions in the Drop Jump trials due to improper landings, yet otherwise ensured comprehensive inclusion. In general, the majority of studies demonstrated a high level of transparency. However, some studies could benefit from enhancing the clarity of their participant inclusion criteria.

The studies utilized varied reference standards, demonstrating varying degrees of methodological rigor. Several studies employed well-established methodologies for motion analysis, including the Vicon Motion System (21, 23), while others utilized force plates (24, 25) to assess the validity and reliability of their mobile systems. These methods are regarded as highly accurate and provide an excellent reference for motion data. Conversely, alternative reference standards have been employed in other studies. While these standards are not regarded as gold, they are nonetheless based on biomechanical rules. For instance, Li et al. (27) used the baseball swing as a reference standard, while Jeon et al. (26) employed fitness exercises. These approaches provide a reasonable basis for comparison when evaluating the performance of the mobile systems despite the potential for less accurate results compared to the gold standards. Fernandez et al. (24) and Tran et al. (21) conducted simultaneous tests with the reference standard, whereas Stanton et al. (25) tested the MyJump application under controlled conditions. Fanton and Harari et al. (20) validated the Amazon Halo movement using standardized conditions, and Aoyagi et al. (23) tested their smartphone application in conjunction with the reference standard. These methodological approaches ensured stable conditions and supported result accuracy.

The discrepancies in study design, participant recruitment, and the utilization of reference standards across these studies underscore the inconsistencies in study quality that impact the results’ internal validity, reliability, and generalizability.

### 3.5 Usability and motivation in camera-based movement screening apps

A key factor for the long-term acceptance and effectiveness of camera-based movement analysis applications is their usability. This includes not only intuitive navigation and ease of use but also the clarity of feedback and comprehensibility of exercise instructions. Studies such as that by Jeon et al. (26) show that real-time feedback is only effective when presented in a clear and immediately actionable way. Their mobile system combines visual feedback with a standardized movement database, enabling users to instantly recognize how their movement deviates from the ideal form—a form of usability that encourages active engagement with one's own movement behavior. Pham et al. (22) likewise emphasize that the combination of a user-friendly graphical interface and precise motion evaluation at both the frame and sequence level is essential for app acceptance. The provision of clearly structured scores and feedback allows users to track their training progress in a transparent and motivating way. In addition, motivation plays a central role in user retention. The Halo Movement app (20) uses a scoring system based on various functional movement tests that culminates in a comprehensive and easy-to-understand overall score. This score enables users to monitor their “movement health status” over time and document improvements—a motivational mechanism that encourages regular engagement with the application. The authors also point out that for healthy adults in particular, the preventive nature of such feedback is often perceived positively and reinforces continued use. However, several studies also revealed that poor usability—for example, due to complex menu navigation, unclear feedback, or limited customizability—can hinder both usage frequency and overall effectiveness [e.g., (23)]. Future development should therefore prioritize adaptive user interfaces and personalized feedback systems to foster high user engagement and sustained adherence.

## 4 Discussion

The rapid expansion of mobile applications within the health and fitness industry has facilitated enhanced access to health monitoring tools (29). This growth is indicative of two major factors: significant advancements in technology and a pervasive shift towards self-management in health and fitness. This trend is further fueled by consumer demand for customized, accessible solutions (30). As mentioned in the introduction, validating these mobile applications is imperative, particularly in movement screening. This systematic review aims to provide a comprehensive overview of camera-based mobile movement screening applications in healthy adults. It focuses on these applications' particular skills and capabilities and examines their accuracy and limitations.

### 4.1 Comparison with established methods and technological challenges

Optical 3D motion capture systems are regarded as the gold standard in motion analysis and are renowned for their precision. Nevertheless, these systems' high cost and complexity have primarily restricted their use to clinical and research settings, severely limiting access to the general population (16). In contrast, smartphone-based systems offer a more economical and available option (31). The question of the extent to which this low-cost, app-based alternative is comparable to established methods gives rise to the following points for discussion. The applications still require enhanced sensitivity to precisely detect fine-motor movements, such as those of smaller body parts (21). Furthermore, technical challenges must be considered, including but not limited to optimal camera positioning. Moreover, mitigating movement disturbances under real-world conditions is imperative to ensure the desired level of precision (25). Standardization is required to ensure validity and reliability (20). Moreover, the question of generalizability to more complex movement patterns remains unanswered (24). These challenges highlight the need to improve the precision and validity of mobile applications, particularly in dynamic movement contexts where accuracy tends to decline significantly (32). This underscores the importance of systematically evaluating their reliability under real-world conditions and across various devices [see also (33)]. Despite these limitations, the accelerated evolution of mobile technologies suggests that smartphone applications for motion analysis have the potential to surpass traditional marker-based systems in terms of efficiency in the future.

### 4.2 Pose estimation frameworks and movement-specific reference standards

The reviewed applications employ a variety of pose estimation frameworks, each of which affects both the functional scope and the validity of movement assessment. OpenPose, as used by Li et al. (27), and TensorFlow-based real-time estimation networks (26) serve as the backbone for applications focused on immediate movement correction and performance monitoring. These models allow for dynamic feedback and deviation detection during exercise execution, making them particularly relevant for qualitative movement screening or coaching-oriented scenarios. In contrast, MediaPipe, as implemented by Pham et al. (22), is primarily used for static joint angle classification and activity recognition. However, due to the absence of an external reference standard or biomechanical validation, the interpretability of the reported accuracy metrics remains limited. As such, MediaPipe-based applications may be better suited for informal fitness guidance rather than structured movement diagnostics. Aoyagi et al. (23) introduced a distinct approach with their custom-built TDPT-GT framework. This system utilizes a deep learning model trained on synthetic motion data to enable three-dimensional, full-body gait tracking. While promising in scope, the use of synthetic training data and the lack of external benchmarking currently restrict its applicability in clinical or high-performance sports contexts.

Importantly, due to the diversity of movements assessed, different reference standards were applied across studies—further limiting comparability. For instance, force plates were used as the reference standard in counter-movement jump analyses (25), while Vicon motion capture systems were employed in gait assessment (21, 23). These methodological differences underscore the need to interpret psychometric properties—such as validity and reliability—within the specific context of the movement and its corresponding measurement standard.

### 4.3 Feedback

Absent direct feedback, users cannot ascertain the accuracy of a movement recorded on a smartphone. It is also crucial to differentiate between the various forms of feedback (34). A simple binary score like “correct” or “incorrect” is inadequate for providing constructive feedback. It fails to inform users which aspects of their performance need improvement or how to execute the movement more effectively (34). Moreover, a binary score merely indicates the extent to which a movement deviates from the ideal execution, providing no specific guidance on correcting or improving the performance (34). Therefore, real-time coaching has been shown to offer several immediate advantages, particularly in maintaining the quality of movement and reducing the risk of injury, primarily when users perform the exercises independently (34). The provision of direct feedback has been demonstrated to play a pivotal role in the prevention of erroneous movements that have the potential to result in injury. Additionally, it has the potential to alleviate pressure on overloaded healthcare systems by reducing healthcare costs (34, 35). However, providing real-time feedback is contingent upon a robust technological infrastructure and a well-designed user interface, which is necessary to achieve the aforementioned benefits. A salient finding is the paucity of personalized feedback in most reviewed applications.

Pham et al. (22) and Jeon et al. (26) were demonstrating the potential for real-time correction. Pham et al. (22) employed MediaPipe to analyze individual joint movements and provided users targeted feedback for improving performance. Jeon et al. (26) implemented a 2D pose estimation system that was integrated with an action database, facilitating real-time detection of deviations and corrective feedback. Both studies were notable for using real-time pose estimation technologies, structured movement evaluations based on established criteria, and comprehensive joint-specific motion analysis. The MyJump app delivers instant feedback by displaying the achieved jump height after each jump, giving athletes direct performance insights (25). Similarly, Amazon's Halo Movement app provides an immediate movement quality score upon completing a movement screening (20). Of the eight applications included, three systems provide direct feedback on movement execution according to current data. While MyJump and Halo Movement display characteristic values such as jump height or movement score immediately after the action, Jeon et al. (26) even provide camera-based real-time feedback through movement detection and deviation analysis during the exercise. Other systems such as CRapp or the framework developed by Pham et al. (22) also offer feedback, but based on downstream analysis and not in real time. In contrast, the remaining applications either deliver their analysis with a delay after the activity or solely record data without offering any live guidance. The Calf Raise App (CRapp), for example, uses computer vision to automatically measure metrics such as repetition count and lift height during a calf raise test (24). However, it lacks interactive real-time corrections or personalized feedback for the user. Overall, many of the reviewed applications did not incorporate personalized real-time feedback mechanisms, emphasizing the need for further advancements in this area.

### 4.4 Population

Except for Fanton and Harari et al. (20), the included studies had a limited sample of participants. As indicated by the findings in Table 2, most of the extant studies exhibited inadequate sample sizes, raising concerns regarding the generalizability of the results. Sample size requirements should be aligned with the study's objective. As Sim and Wright (36) emphasize, studies assessing reliability or agreement require adequate sample sizes based on statistical criteria, such as the expected level of agreement. Tools like G*Power (37) offer practical guidance for power analysis in planning such studies, particularly in validation contexts. This highlights the importance of tailoring sample size to methodological rigor and study design.

## 5 Limitations

In addition to the constraints inherent to the studies included in this review, this analysis is also subject to certain limitations. As delineated in the present analysis, incorporating an additional search term, such as “direct feedback” or “user experience,” could have been a valuable addition during the review process of the articles. However, this may have resulted in an even lower number of hits. A further limitation of this review is the exclusion of studies dealing with children and adolescents. This exclusion may have reduced the number of studies identified, and more applications could have been analyzed. This exclusion may have reduced the number of studies identified, and more applications could have been analyzed. Additionally, the absence of a quality assessment checklist poses another limitation, as no suitable checklist was available. Developing customized quality assessment instruments that address the particular requirements of interdisciplinary studies in movement science would be advantageous for future systematic reviews. In order to facilitate more consistent evaluation and comparability across studies, it is imperative that these tools consider diverse study designs and technical criteria, including but not limited to sensor accuracy, real-time feedback, and usability. Also, an important aspect of this review is its focus on applications that have been examined in peer-reviewed studies. As a result, not all potentially relevant applications currently available on the market were included. The absence of certain applications does not necessarily reflect low quality, but rather a lack of published evidence. This highlights the need for future research to expand and empirically evaluate newer, widely used applications in order to assess their effectiveness, accuracy, and usability

## 6 Conclusions

Despite the growing potential of camera-based smartphone applications for movement analysis in healthy adults, this systematic review reveals that their scientific validation remains limited and heterogeneous. While some studies demonstrated methodological transparency and high scientific rigor, others exhibited notable weaknesses that compromise the reliability of their findings. Of the eight included applications, only three – MyJump, Halo Movement, and the system developed by Jeon et al. (26)—provided camera-based real-time feedback, a key component for effective movement coaching. The remaining applications record motion data reliably but do not deliver immediate feedback during exercise execution. For readers, this review offers tangible practical value: it provides evidence-based insight into which applications have been scientifically validated, which types of movement they address, and whether they offer real-time user feedback. This makes the review a valuable decision-making resource for professionals in sports, therapy, and public health, as well as for developers of digital health technologies. At the same time, there is considerable room for improvement in terms of measurement accuracy, user-friendliness, and the range of movement patterns analyzed. Advances in both hardware and software—particularly more powerful smartphone cameras and more precisely calibrated algorithms—may significantly enhance precision in the future (32). Their low cost and wide availability render smartphone-based applications especially valuable tools in rural or underserved areas where access to conventional sports and health infrastructure is limited (38). However, successful integration of camera-based movement analysis into existing prevention, therapy, or training programs requires close collaboration between app developers, healthcare professionals, and sports scientists. The long-term success of such applications depends on high accuracy in movement detection, clear and comprehensible instruction of the required movement, and sustained user engagement supported by motivational, adaptive, and user-friendly (i.e., high-usability) features.

## 7 Future research

Future research is required to enhance the accuracy and versatility of smartphone-based screening applications in various everyday settings for diverse population groups and a range of movements. Evaluating the agreement between new measurement tools and established gold standards using appropriate methodologies, such as Bland-Altman analysis, is essential since correlation alone is insufficient for establishing equivalence (39). A critical aspect of this process involves the meticulous examination and refinement of well-documented sources of error, such as the positioning of camera equipment and the prevailing lighting conditions. The applications should be designed to be user-friendly and made accessible to any individual with a mobile device. Furthermore, extending the range of exercises to encompass a more diverse array of movement sequences is essential. Moreover, feedback mechanisms must undergo revision and integration to facilitate real-time corrections, thereby effectively minimizing erroneous movements. The incorporation of artificial intelligence (AI) into the feedback mechanisms has the potential to provide customized, adaptive feedback based on individual user movement patterns over time. Additionally, augmented reality overlays may offer intuitive, real-time visual guidance by superimposing ideal movement trajectories directly onto the user's body. These technologies are promising for enhancing user engagement, learning, and injury prevention in future applications. Although this review did not address user experience and engagement, these factors are critical for the long-term application of these technologies. Ensuring a positive user experience and maintaining user engagement is imperative to guarantee these technologies' long-term success. In this context, sustainability should be a key consideration in developing and deploying such applications. Moreover, beyond the validation of newly developed applications, future research should emphasize long-term and intervention-based studies to determine whether these tools are truly effective and beneficial in real-world settings over time. The terms “user experience,” “motivation,” and “usability” were not included in the search criteria, which represents a limitation of our study. In addition, ethical and privacy considerations will be an important factor in the future development of these technologies. The collection and analysis of sensitive individual movement data via smartphones pose privacy risks that must be addressed by implementing appropriate regulations and data security measures (40).
